# P-1859. Immunity against Acute Human Babesiosis and Lyme Disease

**DOI:** 10.1093/ofid/ofae631.2020

**Published:** 2025-01-29

**Authors:** Rimanpreet Kaur, Arshia Arasappan, Rudline G Zamor, Brigitte Maczaj, Victoria A Bateman, Sarath Nath, Luis A Marcos, Charles K Vorkas

**Affiliations:** Stony Brook University, Stony Brook, New York; Stony Brook University/Department of Microbiology and Immunology, Stony Brook, New York; Stony Brook University Hospital, Stony Brook, New York; Stony Brook Medicine, Stony Brook, New York; Stony Brook University, Stony Brook, New York; Stony Brook University Hospital, Stony Brook, New York; Renaissance School of Medicine at Stony Brook University, Stony Brook, New York; Stony Brook University, Stony Brook, New York

## Abstract

**Background:**

Babesiosis,a disease caused by *Babesia microti (Bm) and* transmitted by *Ixodes scapularis* ticks, has significant morbidity in older and immunocompromised hosts. The natural host response against *Bm* has not been well-studied. Co-infection with *Borrelia burgdorferi* (*Bb*), the etiology of Lyme disease (LD) transmitted by the same deer tick, may impact clinical severity. We aimed to define the immune response to acute human babesiosis and LD in an endemic region in NY, with the hypothesis that specialized innate lymphocytes act early to control primary *Bm* infection.

**Figure 1.**
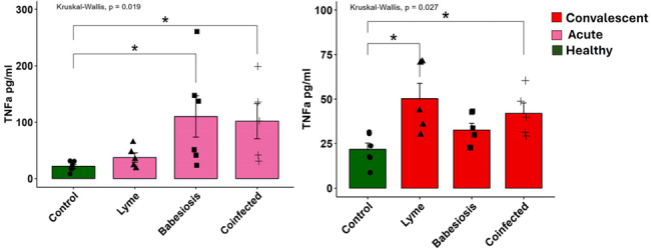
TNFα cytokine profiling for paired acute and 1-month post-treatment cases of babesiosis, Lyme Disease, and Co-infection. n=6/group. Statistical comparison by Kruskal-Wallis test *p<0.05

**Methods:**

Serum and Peripheral Blood Mononuclear Cells (PBMCs) were isolated from a well-characterized human cohort study: acute hospitalized study volunteers with confirmed *Bm* by peripheral blood microscopy and PCR (n=13), *Bb* infection by CDC criteria (n=5) or *Bm/Bb* co-infection (n=5) and compared to paired samples 1-month post-infection or healthy controls (n=12) collected during 2021-2023 at Stony Brook University Hospital, Long Island, NY. PBMCs from babesiosis cases were analyzed by flow cytometry and serum cytokine profiling was performed in mono/co-infected cases to define immune responses over time.

**Figure 2.**
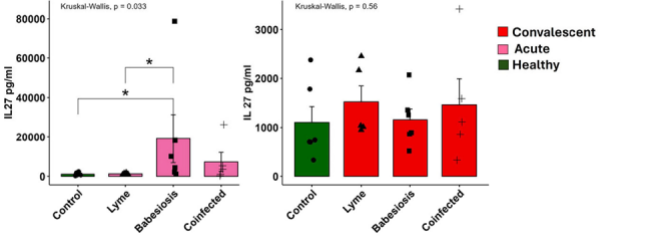
IL27 cytokine profiling for paired acute and 1-month post-treatment cases of babesiosis, Lyme Disease, and Co-infection. n=6/group. Statistical comparison by Kruskal-Wallis test *p<0.05

**Results:**

Acute monoinfected babesiosis cases demonstrated inflammatory signaling through TNFα and IL27 while IL6, IL21, and IL10 were elevated in *Bm* and *Bb* mono/co-infection. Cytokine signatures trended toward resolution one-month post-*Bm* infection, but were persistently elevated during LD. We also observed persistently elevated CXCL10 and TNFα in LD. Flow cytometry of babesiosis cases vs controls revealed expansion of activated innate lymphocyte subsets including CD8α^+^ γδ T cells and Natural Killer (NK) cells. In contrast, innate T cells called MAIT cells were activated, but depleted from blood. There was no difference in the frequency of B cells, conventional CD4^+^/CD8^+^ T cells, or regulatory T cells during infection.

**Figure 3.**
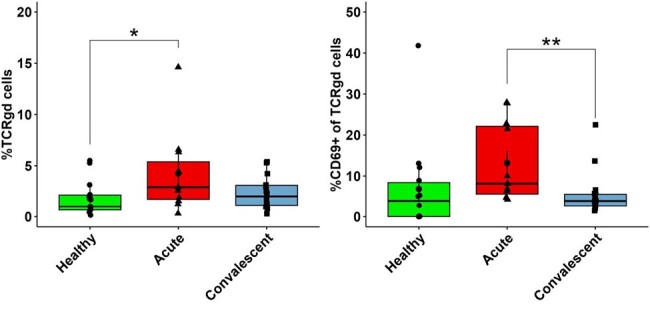
The frequency and activation of γδ T cells in paired acute and 1-month post-treatment cases of babesiosis. n=12 healthy, n=13 babesiosis; Comparisons by Kruskal-Wallis test *p<0.05 **p<0.005

**Conclusion:**

This study identifies distinct inflammatory pathways underlying acute human babesiosis and LD and highlights early immune responses of innate lymphocytes during acute *Bm* infection. Ongoing studies will define the immune mechanisms that control primary *Bm* infection and will determine how this immunity is modulated by *Bb* co-infection.

**Figure 4.**
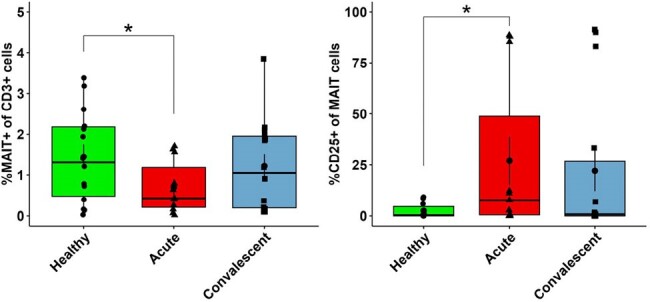
MAIT cell frequency and activation in paired acute and 1-month post treatment cases of babesiosis. n=12 healthy, n=13 babesiosis; Comparisons by Kruskal-Wallis test *p<0.05

**Disclosures:**

All Authors: No reported disclosures

